# Occupational Asthma Prevalence among Migrant Workers Attending Shuaiba Industrial Medical Center in Kuwait

**DOI:** 10.3390/healthcare11142021

**Published:** 2023-07-13

**Authors:** Hussah Waleed Alhadlaq, Alanoud Ateeq, Abdulaziz M. F. Shayea, Janvier Gasana

**Affiliations:** 1 Department of Environmental and Occupational Health, College of Public Health, Kuwait University, Safat 13110, Kuwait; 2 Shuaiba Medical Industrial Center, Occupational Health Department, Ministry of Health, Ahmadi 47005, Kuwait; 3 Departments of Occupational Therapy, Faculty of Allied Health Science, Kuwait University, Kuwait City 12037, Kuwait; 4 Departments of Molecular Biology, Faculty of Graduate Studies, Kuwait University, Kuwait City 12037, Kuwait

**Keywords:** bronchial asthma, industry, migrant workers, Kuwait

## Abstract

Specific work environments, such as exposure to chemicals emitted during industrial processes, are related to occupational asthma. From 1985 to 2012, Kuwait was expected to have the highest asthma prevalence rate among Middle East nations, at 15%. This cross-sectional study was conducted using secondary data from occupational health physicians’ records in the Shuaiba Industrial Medical Center (SIMC) extracted and analyzed using SPSS. Chi-square test and logistic regression were used to check the association between risk factors and bronchial asthma (BA). The data sample size was 3478 in 2018 and 3807 in 2019. In 2018, BA had a significant relationship with age categories, work year groups, and determinants of fitness. Migrant workers above 51 years of age had a high risk of developing BA (*p*-value = 0.012). There was a high risk of developing BA in workers who worked > 21 years (*p*-value < 0.001) and in workers who worked between 11 and 20 years (*p*-value = 0.042). Overweight workers had a risk of developing BA (*p*-value = 0.042). In 2019, BA had an associated relationship with age categories and determinants of fitness. Workers above 51 years of age had about a 39% risk of developing BA (*p*-value = 0.009). Otherwise, the BMI, working year groups, marital status, and smoking status had no association with BA. In conclusion, BA is prevalent among migrant workers at the SIMC. Long hours, low income, and a lack of PPE are just a few of the issues that migrant workers have been exposed to, raising their risk of poor health.

## 1. Introduction

Chronic airway inflammation, known as bronchial asthma, causes the airways to constrict, making breathing difficult [1]. Exposure to substances or chemicals can lead asthmatic patients to develop asthma attacks or exacerbations [2]. These exposures cause immune cells to develop lung inflammation, which can be life-threatening [3]. According to the World Health Organization (WHO), 15 million disability-adjusted life years (DALY) are lost globally each year, and asthma is responsible for 250,000 deaths [4].

Chemicals such as petrochemicals, polyaromatic hydrocarbons, formaldehyde, chlorine, ammonia, nitric oxides, isocyanates, acid anhydrides, and metals (metal salts) released during industrial operations can cause occupational asthma [5]. In several studies, occupational asthma is considered one of the significant and common forms of work-related respiratory diseases in different countries, accounting for between 9 and 15% of asthma cases in adults [6].

Based on prior studies, occupational asthma can be divided into two categories: sensitizer-induced OA (SIOA), which accounts for 90% of the cases [7], and irritant-induced OA (IIOA), which represents 10% of the cases [8,9]. More than 400 OA-causing agents have been reported, with new causative agents that are added each year to a fast-growing list that is quickly out of date and never complete [10].

The most prevalent chemicals and substances that are found in the oil industry include particular matter (PM), nitrogen dioxide (NO_x_), carbon monoxide (CO), hydrogen sulfide (H_2_S), and sulfur oxide (SO_2_). In particular, refinery emissions include large amounts of hydrocarbons in addition to methane [11]. Exposure to PM causes lung dysfunction. To be more precise, it causes lower airway inflammation and upper airway irritation [12]. Nitrogen dioxide (NO_x_) causes severe respiratory illnesses [13].

Worldwide, the prevalence of asthma is not only increasing due to genetic factors, but lifestyle and environmental risk factors may also be potential causes of the increment in the prevalence of asthma [14]. In the last ten years, the prevalence of asthma and allergy has risen, especially in Westernized countries [12,15]. Several studies indicated that exposure to some substances in the workplace could be the cause of more than 10% of all incidents of asthma cases [16,17].

Moreover, data illustrate that asthma prevalence varies across Middle East countries and within a single country’s cities [18]. Kuwait had the highest asthma ranking, with a total of 15% among adults estimated by a self-designed questionnaire. In addition, Tehran had the lowest prevalence, with an estimated 2% from 1985 to 2012 [18,19].

In Kuwait, the data of 2019 showed that 70% of the total Kuwait population is non-Kuwaiti. More than 60% of the non-Kuwaiti population is migrant workers. Most of them are from South and Southeast Asia, such as Afghanistan, Pakistan, India, Nepal, Bangladesh, and Sri Lanka [20]. The asthma prevalence in migrant workers’ parent countries was estimated to be 5.2% in Bangladesh, 6.3% in India, and 4.2% in Nepal. In addition, in Pakistan, it was 3.7% and 5.3% in Sri Lanka [21].

Asthma prevalence has been increasing among workers in various industries, including oil companies in Kuwait, due to exposure to the emission of harmful substances. The amount and period of exposure to these toxic materials need to be addressed and assessed in order to protect these workers from health issues. In this proposal, data from workers who attend the Shuaiba Industrial Medical Center (SIMC) will be assessed, and a recommendation will be drawn to prevent asthma that is caused by exposure to the substances which are associated with asthma.

## 2. Study Hypothesis

Prolonged exposure to harmful chemicals and substances emitted from various industries in Kuwait may be associated with the development and/or exacerbation of asthma among migrant workers. Other variables might also contribute to speeding up this development and/or exacerbation of asthma, such as age, exposure time, other health problems, worker experience, etc.

## 3. Purpose of the Study

The cumulative evidence reviewed demonstrates a consistent lack of estimating occupational asthma among migrant workers in various parts of the world. However, the level of asthma prevalence among migrant workers in the state of Kuwait has not been estimated. The authors of this study strongly believe that exposure to harmful chemicals and substances is the first and most important initial step from which to start investigating occupational asthma among migrant workers. Therefore, the purposes of this study were to estimate the prevalence rate of occupational asthma among migrant workers who attend the Shuaiba Industrial Medical Center in the state of Kuwait; investigate the prevalence of possible risk factors, which are age, exposure time, other health problems, worker experience in Kuwait petrochemical industry, rank, and position; and evaluate the association of asthma and the exposure to harmful chemicals and substances to draw out a prevention recommendation.

## 4. Materials and Methods

### 4.1. Study Design

The Shuaiba Industrial Area in Kuwait served as the site of this cross-sectional investigation. Secondary data from Shuaiba Industrial Medical Center (SIMC) were obtained for the years 2018 and 2019, and the data analysis process commenced in November 2020 and ended in March 2021. Migrant workers from various industrial factories and corporations located in the Al Ahmadi governorate are served by the SIMC [22]. However, Al-Ahmadi is one of Kuwait’s six governates. Thus, the SIMC is responsible for the workers who are employed in the Al Ahmadi governorate. Moreover, this Medical Center serves all the migrant workers from different industrial factories and corporations, including constructors, refinery workers, and agricultural workers in the Al Ahmadi governorate area. Therefore, the SIMC oversees the personnel employed by the Al Ahmadi governorate. However, each governate has a healthcare center that is in charge of caring for its citizens.

### 4.2. Procedure

Secondary 2-year data on asthma were used in this study, and the authors and SIMC occupational physicians examined the data to verify if the data were appropriately collected. The data are a retrospective record that is protected in terms of privacy and confidence. We retrospectively retrieved the secondary data and analyzed these data that were collected during the medical surveillance. We obtained permission from the Ministry of Health MOH to analyze these data. Ethical approval to conduct this study was received from both MOH and Kuwait University—Health Science Center Committees. The ethical approval number is 1658. As the record only contains data that were anonymized, the workers’ information will be protected and confidently secured.

### 4.3. Instruments

Pre-employment examination, periodical examination, confined space entry, and baseline health examination records are among the data-gathering tools used by SIMC’s occupational physicians to assess the prevalence of asthma among migrant workers. The portion of these exams that fall into each category is shown in Table 1. Asthma cases are actually diagnosed by clinical examination, chest x-ray, and using spirometer test. Chest X-ray was performed by using portable X-ray machine, which was used in the workplace, whereas spirometer test was used according to GINA guidelines [23]; participants were asked to perform forced expiratory maneuver where three reproducible curves were obtained (±5%). The spirometry was performed from 8 to 11 am. The measured parameters included FVC, FEV1, FEV1%, and PEFR [24]. There was no exclusion. These tests were performed for all industrial workers in the area. All industrial workers, who visited the SIMC, did all of these routine tests. These tests were used to diagnose occupational asthma by occupational physicians. The diagnosis of asthma is based on the recognition of a characteristic pattern of symptoms and signs and the absence of an alternative explanation for them. The key is to take a careful clinical history. The likelihood of an asthma diagnosis increases where symptoms include at least two of wheezing, breathlessness, chest tightness, and cough; auscultation reveals a widespread wheeze; and there is an otherwise unexplained low forced expiratory volume in 1 s (FEV_1_) or peak expiratory flow (PEF) [25]. Moreover, SIMC collects data from medical fitness tests, which were performed by occupational physician. Fitness determents are divided into three categories: fit, unfit, and fit (FSC). A worker who is physically fit for any work is considered fit. A worker is considered to be unfit if they are unfit or unsafe to do any work due to severe health conditions such as advanced heart disease with threatened failure and malignant hypertension. A worker with a health condition such as defective hearing, hypertension, or diabetes that is still fit to work is considered FSC [26]. In addition, experts at the Integrated Medical Center assigned the irritant exposures and reported them in secondary data.

### 4.4. Data Analysis

The SPSS software program version 26 was used for both data entry and statistical analysis of the data [27]. Statistical analysis of the results included both descriptive and analytic techniques. Descriptive statistics, including the mean, standard deviation, percentage, and frequency, were used to describe personal and sociodemographic characteristics among the study population. The BMI categories were underweight—BMI under 18.5 kg/m^2^, normal weight—BMI greater than or equal to 18.5 to 24.9 kg/m^2^, overweight—BMI greater than or equal to 25 to 29.9 kg/m^2^, obesity—BMI greater than or equal to 30 kg/m^2^, and extremely obese class I—BMI 30 to 34.9 kg/m^2^ [28]. Analytic measures included a two-sample *t*-test and a Chi-square test. A correlation matrix was used to assess the degree of association between different variables, including working year experiences and other independent variables. A logistic regression model was used to describe the diagnosis of asthma and test more than one independent variable in their prevalence between the studied groups after adjusting for age, smoking habit, and exposure. A level of *p* ≤ 0.05 was considered to be statistically significant. Data multiple imputation method was used to consider missing data. We created a five-imputation dataset. It aims to allow for the uncertainty about the missing data by creating several different plausible imputed datasets and appropriately combining results obtained from each of them. The first stage was to create multiple copies of the dataset, with the missing values replaced by imputed values. The second stage was to use standard statistical methods to fit the model of interest to each of the imputed datasets. Standard errors were calculated using Rubin’s rules [29], which take into account the variability in results between the imputed datasets, reflecting the uncertainty associated with the missing values. Multiple imputation was used because it had the potential to improve the validity of medical research.

## 5. Result

### 5.1. Descriptive Analysis of Demographic Characteristics

Table 2 presents the characteristics of 3478 industrial workers who visited the SIMC in 2018 and 3807 industrial workers who visited the SIMC in 2019. The age range of the participants was 21–77 years, with a mean age of 40 years. Moreover, the worker’s experience years in Kuwait’s petrochemical industry ranged from 3 to 52 years, with a mean of 7 years. In addition, the score range was 14.98–51.39, with a mean of 27.19. However, the BMI categories were based on the general classification. The BMI categories are underweight, normal weight, overweight, obese, and extremely obese.

Moreover, according to the 2019 data, the age range was 20–72 years, with a mean of 39 years of age. The worker’s experience years were 7 years in the Kuwait petrochemical industry, with a range of 2–62 years. In addition, the score range of BMI was 15.15–50.38, with a mean of 26.98.

In the 2018 data, Indians made up the largest percentage of migrant workers (56.8%), followed by Egyptian (13.9%), Bangladeshi (7.8%), and Filipino (5.2%) workers. Most of the workers (80%) worked less than 10 years; 16.2% of the workers had a tenure of employment between 11 and 20 years; 2.7% of the workers had worked for more than 21 years.

Furthermore, the majority of the workforce in the Shuaiba area was made up of 31–40-year-old workers, followed by 41-to-50-year-old workers. At that time, 20.5% of the workers were between the ages of 21 and 30, while just 16.5% of the workers were above the age of 51. Additionally, 49.1% of the migrant workers were overweight, followed by 28.7% of the workers who had a normal BMI, 16.9% of the workers who were obese, and a small percentage of the workers who were extremely obese and underweight.

In addition, the majority of the irritant exposure in the Shuaiba area was made up of chemical/H_2_S at 26.4%, followed by noise, heat, and dust at 14%. At that time, 10.4% of the workers were technicians, while 10.1% of the workers were drivers.

On the other hand, about half of the workers in the 2019 data were Indian (56.4%), followed by workers from Egypt (10.5%), Bangladesh (6.6%), and Kuwait (5.9%). About 59.4% of the workers had tenures of less than 10 years (<10), followed by workers who worked between 11 and 20 years (10.5%) and those who worked more than 21 years, with a percentage of 1.9%.

Workers in the middle age range of 31–40 years were about 35.8%, followed by those between the age of 41 and 50 years with a percentage of 24.8% and those between the age of 21 and 30 years with a percentage of 22.1%. Finally, 17.3% of the workers were above 51 years in the overall age categories. Moreover, the percentage of migrant workers in Shuaiba industrial companies who were overweight was about 34.9%, compared to 21.7% of those with a normal BMI and 12.6% of those who were obese, followed by workers who were extremely obese (2.6%) and underweight workers (0.8%) (Table 2).

### 5.2. Descriptive Analysis and Measuring the Association between Risk Factors and Bronchial Asthma

Table 3 shows that 99.3% of the migrant workers in 2018 were not asthmatic and were non-smokers. Approximately 99.5% of the smokers did not have asthma. The Chi-square test resulted in a *p*-value of 0.527, indicating that there is no association between smoking and bronchial asthma.

In 2018, none of the migrant workers between the ages of 21 and 30 presented with bronchial asthma. Additionally, just 0.4% of the workers in the age range of 31 to 40 years had bronchial asthma, compared to 99.6% of those in that group overall. Further, 99.2% of the migrant workers in the 41–50 age group did not have bronchial asthma, while 0.8% of those in the same age group did. Finally, the percentage of workers over 51 years without asthma was roughly 98.3%, while the remaining workers (1.7%) had the condition. Furthermore, there was a significant association between age and bronchial asthma with an asymptotic significance of 0.001. Additionally, with a *p*-value of 0.997, there was no association between nationality and bronchial asthma. Additionally, BA and the Body Mass Index (BMI) were not significantly associated, having a *p*-value of 0.374.

Furthermore, just 0.4% of the workers (99.6%) who had worked for <10 years reported symptoms of asthma. Additionally, 1.2% of the workers in the same years had bronchial asthma, compared to 98.8% of those who had been employed for between 11 and 20 years. Furthermore, 4.3% of the workers and 95.7% of migrant workers who had worked for more than 21 years had no bronchial asthma. Thus, there was a *p*-value of 0.001, indicating a significant association between the working year groups and bronchial asthma.

In 2018, 100% of fit employees were free of bronchial asthma. Further, 77.8% of the unfit workers did not have bronchial asthma, but 22.2% of the unfit workers had BA. Additionally, 98.7% of workers had a fit specific condition (FSC) but no bronchial asthma, compared to 1.3% of workers with an FSC and bronchial asthma. With a *p*-value of 0.001 for the Chi-square test, there was a significant relationship between fitness and bronchial asthma. However, there are three categories for health fitness: fit, unfit, and fit (FSC). The worker who is physically fit for any work is considered fit. The worker is considered to be unfit if they are unfit or it is unsafe for them to do any work due to severe health conditions such as advanced heart disease with threatened failure and malignant hypertension. A worker with a health condition such as defective hearing, hypertension, or diabetes that is still fit to work is considered to have an FSC.

In 2019, the percentage of migrant workers who did not smoke and did not have asthma was 98.6%. Additionally, 99.1% of the workforce was non-asthmatic and smoked. Asthma sufferers who also smoked made up roughly 0.9% of the population. Furthermore, an asymptotic significance of 0.342 indicates that smoking and asthma do not significantly interact.

Furthermore, according to data from 2019, only 0.6% of workers had bronchial asthma, compared to 99.4% of those in the age range of 21 to 30 that did not have BA. Additionally, just 1.2% of workers in the age group of 31 to 40 had bronchial asthma, while 98.8% of those in that group had no history of the condition. Furthermore, 1.4% of the workers in the 41–50 age range as migrant workers had asthma, compared to 98.6% of migrant workers in the same age group who did not have bronchial asthma. Finally, the percentage of workers over the age of 51 without a history of bronchial asthma was around 97.7%, while the remaining 2.3% had the condition. Age and bronchial asthma are highly associated, with a *p*-value of 0.037. With a *p*-value of 1.000, there was no association between nationality and bronchial asthma. Additionally, a *p*-value of 0.647 indicated that there was no association between BMI and bronchial asthma.

Only 1.1% of workers had asthma, and 98.9% of those who had been employed for less than ten years had no bronchial asthma. Additionally, 2.0% of the workers who had worked for 11 to 20 years had bronchial asthma, compared to 98.0% of those throughout the same working years. In addition, 2.7% of the workers who had been migrant workers for more than 21 years and did not have bronchial asthma made up 97.3% of the total. With a *p*-value of 0.342, the Chi-square test revealed no association between the working year groups and bronchial asthma.

Furthermore, 99.9% of the workers were fit and free of bronchial asthma, compared to 0.1% of the workers who were fit but had the condition. Additionally, the percentage of unfit employees without bronchial asthma was close to 100%. Additionally, 97.4% of workers had a fit specific condition (FSC) but did not have bronchial asthma, compared to 2.6% of workers with an FSC and bronchial asthma. With a *p*-value < 0.001, there was also a significant relationship between fitness and bronchial asthma.

### 5.3. Measuring the Association between Workload and Bronchial Asthma

With a *p*-value of 0.787, the Chi-square test in 2018 found no association between workload and bronchial asthma. Additionally, 2019 data with a *p*-value of 0.593 also found that there was no association between workload and bronchial asthma (Table 4).

### 5.4. Logistic Regression Test between Demographic Categories and Bronchial Asthma

Regarding the logistic regression model relationship between categories and bronchial asthma, migrant workers who are over 51 years old were correlated with being diagnosed with bronchial asthma in 2018, with a *p*-value of 0.012. Smoking and marital status were also unrelated to bronchial asthma. Furthermore, migrant workers who worked for more than 21 years had a significant risk of developing bronchial asthma (93.1%), while those who worked for between 11 and 20 years had a risk of roughly 26.2%, with a *p*-value of 0.042. Additionally, workers who were overweight had a 0.042 *p*-value probability of developing the disease.

In 2019, based on logistic regression, those above 51 years of age had about a 39% risk of developing bronchial asthma with a *p*-value of 0.009. On the other hand, BMI, working year groups, marital status, and smoking status had no association with bronchial asthma (Table 5).

## 6. Discussion

In Kuwait, being a migrant worker is associated with a high risk of exposure to inhalant toxic chemicals and substances for extended periods of time because they start working at a younger age, which could lead to permanent health problems, including lung changes and disability [30]. In this study, we evaluated asthma, as the cases were not restricted to those thought to be occupational in origin. Studies in this context focus on risk assessment and prevention strategies and approaches that will affect migrant workers’ health.

### 6.1. The Association between Smoking and Bronchial Asthma

According to our findings, there was no association between smoking and bronchial asthma in either 2018 or 2019. It is disputed whether active smoking contributes to the onset of asthma. Prior to the year 2000, several studies found no link between smoking and the prevalence of asthma. According to Vignoud, L. and his colleagues in 2011 [31], it is still unclear whether smoking causes asthma. On the other hand, numerous studies have demonstrated a strong link between smoking and bronchial asthma. Asthma symptoms and occupational exposures were linked in 2017, according to research by R. Abrahamsen and his colleagues [32]. Additionally, Spears, M. showed in 2009 that smoking could cause occupational asthma [33].

### 6.2. The Association between Body Mass Index and Bronchial Asthma

In our study, BMI had no significant relationship with bronchial asthma in 2018 and 2019. However, many previous published studies showed that there is a significant relationship between obesity and overweight and the incidence of asthma, but until today, there has been no clear understanding that the relationship is either due to a causal association or the two conditions sharing the same environmental, behavioral, or genetic influences [34,35,36]. Moreover, the precise mechanism is unknown regarding the association between high BMI and the incidence of asthma [37]. Additionally, Sin, D. and E.R. Sutherland showed in 2008 that high BMI was a significant risk factor for the incidence of bronchial asthma [38]. However, we found that there was a significant association between age categories and BMI in both years. In 2018, Dunn, R found that the relative risk associated with higher BMI decreases with age [39].

This study found that people who were less than 50 years of age were likely to have good fitness levels, while people above 50 years of age were at risk of having lower fitness levels. In our results, the association between fitness level and bronchial asthma was significant. However, further studies should be carried out to assess the association between fitness level and bronchial asthma.

### 6.3. The Association between Workload and Bronchial Asthma

This study revealed a strong association between work experience duration and bronchial asthma in 2018. Additionally, there is an association between the duration of work experience and bronchial asthma, according to Rönmark, E., who aimed to establish evidence-based diagnostic and treatment guidelines for work-related asthma [40]. Future studies should also be conducted to establish a clearer correlation between work experience duration and bronchial asthma.

### 6.4. The Association between Industrial Toxic Chemicals and Bronchial Asthma

Several studies showed that good occupational hygiene practice dictates that methods such as material substitution, ventilation, isolation, and other engineering controls should be considered [41]. Moreover, many studies have indicated a significant relationship between asthma and occupational exposure [42,43]. Vlahovich, K published a paper in 2021 that showed an association between asthma and toxic chemical exposure in the workplace, and multiple respiratory disorders, including asthma, have a significant occupational impact that has clinical, policy, and research ramifications [44]. Moreover, in 2018, Zivadinovic, N carried out a study, which was a population-based sample (n = 7120) of participants aged 16–55, from the Telemark study, with a five-year follow-up. By using logistic regression and adjusting for gender, age, education level, and smoking, it was possible to determine the relationship between newly developed asthma and self-reported occupational exposure to vapor, gas, dust, and fumes (VGDF). It showed that there is an association between occupational exposure and new-onset asthma [45].

However, it is difficult to protect any worker who must handle low-molecular-weight (LMW) chemicals. Respiratory masks and air-supplied respirators can leak, and many employees find it difficult to put up with uncomfortable safety gear for the duration of a shift. Grammer et al. observed a decreased prevalence of chemical sensitization and OA among workers utilizing respiratory protection equipment compared to those who did not in a cohort of workers exposed to an acid anhydride chemical sensitizer [46]. Fifty-two published studies on the treatment and outcomes of work-related asthma (WRA) were evaluated in a recent systematic review, which found that complete exposure avoidance is more effective than partial mitigation; persistent exposure is likely to exacerbate asthma, and personal protective equipment does not offer full protection. Careful medical supervision is necessary for any intervention intended to reduce exposure to prevent the worsening of the condition [47].

Finally, the multiple imputation method assumes that the missingness of the data is random. This assumption might be a limitation of our inferences; however, we did not evaluate such an assumption using sensitivity analysis because the sample size was large enough (i.e., n = 7294), and the missing data represented approximately only 15% of the total data.

## 7. Limitations

Our study had several limitations, including the sample size in both 2018 and 2019, which was too low to generate reliable findings. The migrant workers face many barriers, including language and cultural barriers. So, their educational level could affect the results from the secondary data we collected from the SIMC. Additionally, selection bias could occur due to migrant workers’ turnover or return to their countries.

It is challenging to estimate the global prevalence of adult asthma in epidemiological studies. There are several causes, including the use of different terminology for asthma symptoms in various languages and variations in how patients report their symptoms depending on their background. Additionally, because doctors practice in various healthcare systems, they have a variety of diagnoses.

Further, it is difficult to draw causal relationships from cross-sectional analysis. However, we can estimate the prevalence of disease and estimate the odds ratios to study the association between exposure and the outcomes in this design.

In this paper, we could not cover all the objectives, but we checked the association between the risk factors and bronchial asthma and evaluated the association between asthma and exposure to harmful chemicals and substances to draw out a prevention recommendation.

It was challenging to explore the main chemicals and substances that might be associated with asthma among these workers, such as particulate matter, nitrogen oxides, and carbon monoxide, etc., because the SIMC did not ask questions about the types of chemicals that the migrant workers work with daily in their jobs. So, in this secondary dataset, no specific chemical was recorded for each migrant worker regarding exposure in their work. We recommend that the SIMC collect data on chemicals that the migrant workers are exposed to while at work. Moreover, there was missing information on the workload for a lot of subjects in 2019.

## 8. Conclusions

More than 60% of the people living in Kuwait are migrant workers. Occupational asthma is associated with a specific work environment and is regarded as one of the most prevalent types of work-related respiratory illnesses in several industrialized nations. Moreover, migrant workers have been subjected to a variety of factors, such as long working hours, low wages, and a lack of PPE, increasing their risk of developing health problems [48].

The results of this study demonstrated that migrant workers who are registered in the SIMC in Kuwait prevalently have bronchial asthma. According to statistics from 2018, there is a significant association between bronchial asthma and various age groups, work year groups, and determinists of fitness. In 2019, there was an association between bronchial asthma and age groups and determinists of fitness.

Future research should concentrate on both primary and secondary prevention to decrease occupational asthma, according to our study’s recommendations. One of the occupational diseases that is difficult to confirm is occupational asthma. Investigations are required to develop better diagnostic techniques that will enable the earlier detection of occupational asthma (OA) and the prevention of future OA cases. Further investigation is advised to examine the relationship between bronchial asthma and years of work experience, in addition to the relationship between bronchial asthma and fitness/worker’s health.

## Figures and Tables

**Table 1:** Portion of the exams fall into each category.

Year	2018		2019	
	Bronchial Asthma		Bronchial Asthma	
	NO	YES	NO	YES
Type Of Medical Check-Up				
Pre-employment	44	1	178	1
Periodical	1293	10	1654	29
Confined space entry	233	3	146	1
Baseline health examination	1847	8	1683	18
medical fitness test (Special Health Medical Examination)	38	0	96	0

**Table 2:** Baseline descriptive analysis of distribution characteristics of industrial workers, SIMC Kuwait, 2018 and 2019.

Years	2018		2019	
n = 3478			n = 3807	
Worker’s Experience Years	Number	Column Total N%	Number	Column Total N%
10>	2787	80.10%	2261	59.40%
11–20	563	16.20%	401	10.50%
21<	94	2.70%	74	1.90%
Missing	34	1.0%	1071	28.2%
**Age Categories**				
21–30 years	712	20.50%	840	22.10%
31–40 years	1270	36.50%	1364	35.80%
41–50 years	921	26.50%	945	24.80%
Above 51 years	575	16.50%	658	17.30%
Missing	0	0%	0	0%
**BMI Categories**				
Under Weight	30	0.90%	29	0.80%
Normal	997	28.70%	828	21.70%
Overweight	1709	49.10%	1329	34.90%
Obese	588	16.90%	481	12.60%
Extremely Obese	152	4.40%	98	2.60%
Missing	2	0%	1042	27.4%
**Nationality**				
Kuwaiti	70	2.0%	223	5.9%
Egyptian	483	13.9%	401	10.5%
Indian	1976	56.8%	2148	56.4%
Bangladeshi	272	7.8%	252	6.6%
Filipino	181	5.2%	144	3.8%
Others	496	14.3%	639	16.80%
Missing	0	0%	0	0%
**Irritants Exposure**				
Ammonia	21	0.6%	3	0.1%
Ammonia, Dust, Noise	7	0.2%	18	0.5%
Ammonia, Natural Gas, and R-22	8	0.2%	13	0.3%
Chemical Material, Dust, General Hazards, and Noise	87	2.5%	116	3.0%
Chemical, Noise, and Vibration	76	2.2%	41	1.1%
Chemical/H_2_S	313	9.0%	1006	26.4%
Chlorine gas and HCl Fumes	213	6.1%	27	0.7%
Dust, Noise, Heat, and UV Rays	19	0.5%	11	0.3%
Crude oil and Dust	35	1.0%	9	0.2%
Dust, Fumes, Gases, Noise, Vibration, and Biological agents	325	9.3%	119	3.1%
Ergonomic, Dust, and Gases	144	4.1%	66	1.7%
Extreme Weather Conditions (Hot, Cold, and Humidity)	19	0.5%	314	8.2%
Eye Strains	9	0.3%	1	0.0%
Fumes and Vibration	1	0.0%	50	1.3%
H_2_S, CO_2_, and SO_2_	476	13.7%	294	7.7%
Hexane, Benzene, Noise, and Dust	1	0.0%	119	3.1%
Hydrocarbon Vapor, Dust	8	0.2%	5	0.1%
Hydrogen Sulfide	5	0.1%	17	0.4%
On-site Dust Exposures	5	0.1%	50	1.3%
Light	1	0.0%	14	0.4%
Paint Vapors	1	0.0%	10	0.3%
Noise, Heat, Dust	618	17.8%	532	14.0%
Noise, Heat, Vibration	231	6.6%	185	4.9%
Office (indoor area)	176	5.1%	6	0.2%
Oil and Gas	1	0.0%	10	0.3%
Smell, Heat, Dust, Chemical Hazards, and General Hazards	84	2.4%	3	0.1%
Static Position	2	0.1%	3	0.1%
Sulfur and Dust	81	2.3%	0	0.0%
Toxic Gases, Noise, and Heat Stress	59	1.7%	57	1.5%
UREA	137	3.9%	0	0.0%
Welding Fumes	2	0.1%	104	2.7%
Water pressure, Mechanical, and Weather	8	0.2%	23	0.6%
Working Open Area	11	0.3%	9	0.2%
Missing	251	7.2%	412	10.8%
**Type of Occupation**				
Physical Scientists and Related Technicians	4	0.1%	8	0.2%
Engineers	139	4.0%	175	4.6%
Surveyors/Draftsman and Assistants	16	0.5%	13	0.3%
Aircraft and Ships Officers	1	0.0%	23	0.6%
Biologists	1	0.0%	4	0.1%
Nurses	16	0.5%	11	0.3%
Statistics/Mathematic/Analysts and Assistants	9	0.3%	24	0.6%
Economists and Accountants	7	0.2%	9	0.2%
Other Professionals/Technical	444	12.8%	397	10.4%
Administrators (Government)	3	0.1%	6	0.2%
Manager (Private Company)	34	1.0%	32	0.8%
Clerical Supervisors	15	0.4%	26	0.7%
Mail/Tel. and Telegraph Operators	4	0.1%	8	0.2%
Other Clerical Workers	105	3.0%	141	3.7%
Sales Supervisors	1	0.0%	4	0.1%
Cooks/Waiters and House Keepers	16	0.5%	25	0.7%
Cleaners	2	0.1%	8	0.2%
Other Service Workers	39	1.1%	51	1.3%
Agriculture and Animal Husbandry Workers	2	0.1%	5	0.1%
Fisherman and Hunters	1	0.0%	7	0.2%
Production Supervisors and Foremen	385	11.1%	345	9.1%
Processors	36	1.0%	66	1.7%
Chemical Processors and Related Workers	16	0.5%	23	0.6%
Carpenter	14	0.4%	26	0.7%
Electrical and Electronic Workers	80	2.3%	106	2.8%
Plumbers	3	0.1%	9	0.2%
Printers and Related Workers	2	0.1%	8	0.2%
Other Production Workers	41	1.2%	95	2.5%
Painter	37	1.1%	50	1.3%
Bricklayers and Other Construction Workers	4	0.1%	36	0.9%
Stationary Equipment Operators	181	5.2%	167	4.4%
Material Handling Equipment Operators	34	1.0%	36	0.9%
Transport Equipment Operators	127	3.7%	142	3.7%
Other Handling Workers (LABOURS)	714	20.5%	609	16.0%
Mechanic	119	3.4%	171	4.5%
Driver	416	12.0%	383	10.1%
Welders	100	2.9%	86	2.3%
Pipe Fitter	38	1.1%	35	0.9%
General Fitter	86	2.5%	68	1.8%
Sailor/Diver	3	0.1%	5	0.1%
Fabricator	43	1.2%	38	1.0%
Sand Blasting Workers	25	0.7%	28	0.7%
Rigger	40	1.2%	32	0.8%
Scaffolder	30	0.9%	26	0.7%
Divers	40	1.2%	58	1.5%
Missing	5	0.1%	182	4.8%

**Table 3:** Descriptive analysis and measuring the association between risk factors and bronchial asthma among migrant workers, SIMC, 2018 and 2019.

Years	2018				2019		
	n = 3478				n = 3807		
	Bronchial Asthma			(*p*-Value)	Bronchial Asthma		(*p*-Value)
		NO	YES		NO	YES	
**Smoking**							
NO	n	2628	18	0.527	3095	43	0.324
	(%)	99.30%	0.70%		98.60%	1.40%	
YES	n	828	4		663	6	
	(%)	99.50%	0.50%		99.10%	0.90%	
**Age categories**							
21–30 years	n	712	0	0.001 *	835	5	0.037 *
	(%)	100.00%	0.00%		99.40%	0.60%	
31–40 years	n	1265	5		1348	16	
	(%)	99.60%	0.40%		98.80%	1.20%	
41–50 years	n	914	7		932	13	
	(%)	99.20%	0.80%		98.60%	1.40%	
above 51 years	n	565	10		643	15	
	(%)	98.30%	1.70%		97.70%	2.30%	
**Working Year Groups**							
<10 years	n	2777	10	<0.001 *	2236	25	0.342
	(%)	99.60%	0.40%		98.90%	1.10%	
11–20 years	n	556	7		393	8	
	(%)	98.80%	1.20%		98%	2.00%	
>21 years	n	90	4		72	2	
	(%)	95.70%	4.30%		97.30%	2.70%	
Missing	n	33	1		1057	14	
	(%)	97.10%	2.90%		98.70%	1.30%	
**Determination of Fitness**							
FIT	n	1798	0	<0.001 *	1861	1	<0.001 *
	(%)	100.00%	0.00%		99.90%	0.10%	
UNFIT	n	7	2		8	0	
	(%)	77.80%	22.20%		100.00%	0.00%	
FSC	n	1518	20		1785	48	
	(%)	98.70%	1.30%		97.40%	2.60%	

FIT = physically fit, UNFIT = physically unfit, and FSC = fit specific medical condition. Note. * Significant differences at the *p* ≤ 0.05 level.

**Table 4:** Chi-square test among workload and bronchial asthma of industrial workers, SIMC Kuwait, 2018 and 2019.

Year		2018				2019			
Variables		Bronchial Asthma			Chi-Square *p*-Value	Bronchial Asthma			Chi-Square *p*-Value
		NO	YES	Total		NO	YES	Total	
Workload category	Low Physical Activity	291	1	292	0.787	275	5	280	0.593
	Moderate Physical Activity	350	1	351		162	1	163	
	High Physical Activity	2815	20	2835		1494	21	1515	
Total		3456	22	3478		1931	27	1958	

**Table 5:** Logistic Regression Test between demographic categories and bronchial asthma, SIMC Kuwait, 2018 and 2019.

Year		2018				2019			
Variables		*p*-Value	OR	95% CI				95% CI	
				Lower	Upper	*p*	OR	Lower	Upper
Age	21–30 years	Reference	Reference	Reference	Reference	Reference	Reference	Reference	Reference
	31–40 years	0.686	1.403	0.272	7.251	1.183	1.982	0.723	5.431
	41–50 years	0.123	2.719	0.563	13.128	1.110	2.329	0.827	6.562
	Above 51 years	0.012 *	6.924	1.528	31.364	0.009 *	3.896	1.409	10.775
Marital status	Single	Reference	Reference	Reference	Reference	Reference	Reference	Reference	Reference
	Married	0.507	1.971	0.266	14.624	0.760	0.874	0.37	2.067
Working Years	Less than 10 years	Reference	Reference	Reference	Reference	Reference	Reference	Reference	Reference
	11–20 years	0.042 *	2.627	1.033	6.679	0.144	1.821	0.815	4.065
	More than 21 years	0.001 *	9.313	2.963	29.276	0.222	2.484	0.577	10.69
BMI	Under Weight	Reference	Reference	Reference	Reference	Reference	Reference	Reference	Reference
	Normal	0.198	0.251	0.031	2.058	0.488	0.482	0.061	3.791
	Over Weight	0.042 *	0.109	0.013	0.926	0.360	0.384	0.05	2.981
	Obese	0.292	0.317	0.037	2.688	0.271	0.294	0.033	2.603
	Extremely Obese	0.403	0.353	0.031	4.055	0.385	0.289	0.017	4.764
Smoking	No	Reference	Reference	Reference	Reference	Reference	Reference	Reference	Reference
	Yes	0.571	0.754	0.283	2.006	0.328	0.651	0.276	1.537

Note. * Significant differences at the *p* ≤ 0.05 level.

## Data Availability

Not applicable.
